# Facility conditions, obstetric and neonatal care practices, and availability of emergency obstetric and neonatal care in 72 rural health facilities in the Democratic Republic of the Congo: A cross-sectional study

**DOI:** 10.12688/gatesopenres.12905.2

**Published:** 2019-07-23

**Authors:** Rebecca Carter, Xu Xiong, Paul-Samson Lusamba-Dikassa, Elvis C. Kuburhanwa, Francine Kimanuka, Freddy Salumu, Guy Clarysse, Baudouin Kalume Tutu, Sylvain Yuma, Alain Mboko Iyeti, Julie H. Hernandez, Jeffrey G. Shaffer, Jane T. Bertrand, Susie Villeneuve, Alain Prual, Lee Pyne-Mercier, Assaye Nigussie, Pierre Buekens

**Affiliations:** 1 Tulane University School of Public Health and Tropical Medicine, New Orleans, Louisiana, 70112, USA; 2 Kinshasa School of Public Health, University of Kinshasa, Kinshasa, Democratic Republic of the Congo; 3 UNICEF-DRC, Kinshasa-Ngaliema, Democratic Republic of the Congo; 4 Ministère de la Santé, Secrétariat général, Kinshasa – Gombe, Democratic Republic of the Congo; 5 UNICEF Western & Central Africa Regional Office, Dakar-Yoff, Senegal; 6 Bill & Melinda Gates Foundation, Seattle, USA

**Keywords:** Clinical mentoring, Democratic Republic of the Congo, emergency obstetric and neonatal care, maternal and newborn health

## Abstract

**Background:** Current facility conditions, obstetric and neonatal care practices, and availability of emergency obstetric and neonatal care (EmONC) were assessed in the Kwango and Kwilu provinces of the Democratic Republic of the Congo ****(DRC).

**Methods: **This is an analysis of the baseline survey data from an ongoing clinical mentoring program among 72 rural health facilities in the DRC. Data collectors visited each of the facilities and collected data through a pre-programmed smartphone. Frequencies of selected indicators were calculated by province and facility type—general referral hospital (GRH) and primary health centers (HC).

**Results: **Facility conditions varied across province and facility type. Maternity wards and delivery rooms were available in the highest frequency of rooms assessed (>95% of all facilities). Drinking water was available in 25.0% of all facilities; electricity was available in 49.2% of labor rooms and 67.6% of delivery rooms in all facilities. Antenatal, delivery, and postnatal care services were available but varied across facilities. While the proportion of blood pressure measured during antenatal care was high (94.9%), the antenatal screening rate for proteinuria was low (14.7%). The use of uterotonics immediately after birth was observed in high numbers across both provinces (94.4% in Kwango and 75.6% in Kwilu) and facility type (91.3% in GRH and 81.4% in HC). The provision of immediate postnatal care to mothers every 15 minutes was provided in less than 50% of all facilities. GRH facilities generally had higher frequencies of available equipment and more services available than HC. GRH facilities provided an average of 6 EmONC signal functions (range: 2-9).

**Conclusions: **Despite poor facility conditions and a lack of supplies, GRH and HC facilities were able to provide EmONC care in rural DRC. These findings could guide the provision of essential needs to the health facilities for better delivery of maternal and neonatal care.

## List of abbreviations

DRC: The Democratic Republic of the Congo

EMEN: Every Mother Every Newborn

EmONC: Emergency obstetric and neonatal care

GRH: General referral hospital

HC: Primary health center

MNH: Maternal and newborn health

ODK: Open Data Kit

UNICEF: The United Nations International Children's Emergency Fund

## Introduction

Maternal and neonatal deaths are a global issue but disproportionately affect low- or middle-income countries. An estimated 99% of maternal deaths occur in low- or middle-income countries, and nearly two-thirds occur in the sub-Saharan African regions ^1^. There are an estimated three newborn deaths per 1,000 live births in high-income countries compared to 27 newborn deaths per 1,000 live births in low-income countries ^1^. Many maternal and newborn deaths are a result of preventable complications during pregnancy and childbirth and immediately after birth. Common causes for maternal deaths include severe bleeding, infections, hypertensive disorders in pregnancy, or delivery complications; for newborns, common causes of death are premature birth, infections, or labor and delivery complications ^1^. Strategies aimed to reduce maternal and neonatal deaths are centered around improving quality and access to antepartum or antenatal care, delivery assisted by a skilled birth attendant, increasing rates of institutional deliveries for better access to intrapartum and postpartum care, and ensuring availability of life saving drugs as well as the good quality of health facility conditions ^1^.

Despite a 30% decrease in maternal mortality since 1990, the maternal mortality ratio in the Democratic Republic of the Congo (DRC) is still among the highest in the world (846 maternal deaths/100,000 live births) ^1, 2^. Similarly, while neonatal mortality has decreased by 33% since 2007, the neonatal mortality rate also remain high (28 newborn deaths/1,000 live births) and contribute to 4% of global newborn deaths ^2^. The DRC boasts one of the highest rates of institutional deliveries in sub-Saharan Africa (80%) ^2^. Additionally, eight out of every ten births also assisted by a skilled provider ^2^. Taken together, this suggests that while availability of health care workers is high, there may be issues with the in-facility quality of maternal and newborn care provided. Therefore, there is a need for continued education for health care providers. To further reduce maternal and neonatal mortality, we are designing and implementing a clinical mentoring program aimed at improving health providers’ skills, knowledge, and attitude to provide a better quality of maternal and newborn care in the DRC ^3– 5^.

Many factors can affect healthcare providers’ effective delivery of high quality care at the facility-level; important factors include the professional environment (e.g., structure) and availability of essential drugs and supplies for provision of emergency obstetric and neonatal care ^6, 7^. These basic needs are often lacking in the low-resource settings of sub-Sahara African countries ^7, 8^. Therefore, before implementing a clinical mentoring intervention program to improve quality of maternal and neonatal care, it is important to assess and understand the current facility conditions, obstetric and neonatal care practices, and availability of emergency obstetric and neonatal care in the health facilities. The objective of this paper is to describe the current facility conditions, obstetric and neonatal care practices (skills, knowledge, and attitudes), and availability and use of emergency obstetric and neonatal care (EmONC) among a sample of 72 rural health facilities in the Kwango and Kwilu provinces, DRC.

## Methods

### Setting and health facility selection

This study is an analysis of the baseline survey data from an ongoing project of “Improving the Quality of Maternal and Newborn Health Outcomes through the Development of a Model of Clinical Mentorship in DRC,” including 72 health facilities in the Kwangu and Kwilu provinces of the DRC, funded by the Bill & Melinda Gates Foundation (grant number OPP1144354). For the clinical mentoring project, among the 72 health facilities selected in this study, 48 were assigned to receive clinical mentoring intervention and 24 were assigned not to receive clinical mentoring intervention as a control group by the Ministry of Health and UNICEF in the DRC. After completion of the baseline survey, the Ministry of Health and UNICEF in the DRC will then implement a 18-month period clinical mentoring program in the group of 48 facilities. Process and outcome data on key maternal and newborn health (MNH) indicators will be collected to assess the performance of the clinical mentors and mentees, as well as the clinical mentoring program. Upon completion of the clinical mentoring implementation, we will conduct an endline survey on the same 72 facilities. We will compare MNH indicators collected from the endline survey of the intervention to baseline data, as well as between the groups with and without the implementation of the clinical mentorship program to assess the effectiveness of the clinical mentoring program.

These 72 health facilities were selected by the DRC Ministry of Health and UNICEF to facilitate the implementation of the ongoing clinical mentorship program, taking the following administrative criteria into account:

1) Size of health facility (at least 200 annual deliveries); this number of deliveries was selected to ensure that some adverse pregnancy complications and birth outcomes would occur during the total 6–8 weeks of clinical mentorship in each facility, thereby allowing the mentor to address some severe maternal and newborn conditions with the mentees.
2) Coverage of health zones (the majority of the health zones include at least one GRH and its affiliated HCs);
3) Accessibility (e.g., ability to reach selected health facilities with ease);
4) Ease of implementation of clinical mentorship program (e.g., selecting health facilities that are near one another to ease mentor travel).

**Figure 1.**
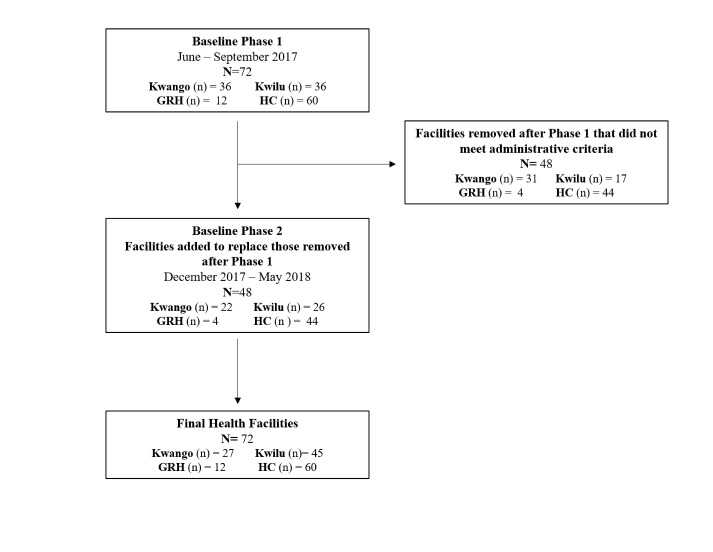
Health facility selection.

For the present analysis of the baseline survey data, the final 72 facilities were a combination of two phases of baseline data collection. The first phase of data collection was conducted June–September 2017 and the second phase of data collection was conducted from December 2017–May 2018. Forty-eight facilities were removed by UNICEF and the DRC Ministry of Health after baseline Phase 1 because they did not meet the first administrative criteria, of more than 200 annual deliveries. Baseline Phase 2 was conducted to replace these 48 facilities with 48 new facilities to have a total of 72 facilities that are included for the ongoing clinical mentorship program ( Figure 1).

### Ethics statement

This project was approved by Tulane Institutional Review Board (IRB) (Reference number: 16-915240) and Kinshasa University School of Public Health Ethics Committee (Approval number: ESP/CE/087/2016).

### Survey formation

To conduct the surveys, the data collectors visited each of the 72 health facilities. During the visits, the data collector used a pre-programmed, smartphone-based application, Open Data Kit ^9^ (ODK Collect v1.16), to complete the following questionnaires:

1) Facility condition form: This checklist includes an assessment of infrastructure, equipment, medicines & supplies, water supply & electricity, and staffing (see Supplementary Table 1).
2) Health providers obstetric and neonatal care practice checklist: This checklist was created based on criteria from Every Mother, Every Newborn (EMEN) Quality Improvement Guide for Health Facility Staff ^10^. Final indicators were determined based on the feasibility to measure these indicators in the field. These indicators cover evidence-based practices during antenatal, labor, delivery, and postnatal care and were used to measure healthcare providers’ skills, knowledge, and attitude during MNH care ^10^. After obtaining informed consent from health care providers, the data collector observed health care providers' routine MNH care practices for ten antenatal care visits and two deliveries at each health facility (see Supplementary Table 2).
3) A 3-month delivery record review and extraction: A data extraction form was created to identify selected maternal and infant morbidity and mortality outcomes (see Supplementary Table 3). When available, data on comprehensive EmONC measures was also collected (e.g., manual removal of the placenta or removal of residual retained products).
4) Emergency Obstetric and Newborn Care Needs Assessment (EmONC): Data on EmONC were collected to assess the availability of Basic (BEmONC) for primary health centers and Comprehensive (CEmONC) for general referral hospitals ^11^. EmONC data was aggregated from the facility condition checklist and three-month delivery record (see Supplementary Table 4).

### Data collection

A total of six native speaking data collectors who were trained medical doctors and had previous research or survey experience were recruited to conduct the baseline surveys. Data collectors were provided with a motorbike for travel to health facilities. Each of the six data collectors visited an average of one health facility per week and stayed at each health facility for 4–6 days. Data collection was completed via questionnaires programmed in Android phones using the ODK system. At each hospital or clinic, all healthcare providers who perform maternal and neonatal care were deemed eligible to participate regardless of their presence in the facility at the time of data collection. Providers who were present at time of data collection were subsequently approached to participate in the survey. Healthcare providers who agreed to participate signed a written informed consent that allowed the data collector to observe their routine maternal and neonatal care practice for ten antenatal care visits and two deliveries; we refer to these unique observations as provider-patient pairs. In addition, the data collectors completed the facility condition assessment and 3-month delivery extraction forms. Upon completion of observations, data collectors submitted the ODK forms to a protected server, where data was aggregated by the research team at the Tulane University Project Coordinating Center in New Orleans, USA.

### Quality assurance and control

Data collectors were trained on how to use the ODK data collection system, how to ride their motorbikes, and how to complete the questionnaires and observations for the selected key indicators prior to the surveys. To ensure a high quality of data collection, a data collection coordination office was established in the capital city of Kikwit in Kwango province. A data coordinator organized and supervised the six data collectors to visit the GRHs and HCs. Hard copies of completed consent forms, delivery records, and questionnaires or facility condition assessment forms were delivered to the Kikwit office when completing the surveys in case there were errors with the ODK system. Additional quality control was managed in two ways: (1) ODK GPS data and server monitoring by the research team in the Tulane University Project Coordinating Center in New Orleans; and (2) weekly Skype calls between the Kinshasa School of Public Health and Tulane University teams to monitor study progress and discuss any issues or challenges raised while fielding the surveys.

### Statistical analysis

***Variables.*** This analysis used quantitative data from the facility condition assessment report. For facility condition data, availability was defined as a combination of medicines, supplies, and physical space that was both available and valid/functional or available and not valid/functional (raw data shown in Supplementary Table 5). Indicators from the antenatal care, labor, delivery, and postnatal care questionnaires were selected that related to EmONC. EmONC signal functions were compiled from the facility condition form based upon availability of supplies (e.g., antibiotics or a newborn ventilation kit were available) as well as the 3-month delivery record abstraction (Supplementary Table 3). Proportions of availability for all measures were generated and summed in order to understand the structural capacity to provide a package of EmONC care.

***Analysis.*** Frequencies and percentages of facility conditions and MNH indicators of antenatal care, labor, delivery, and postnatal care were calculated for the final 72 facilities in aggregate, by province (Kwango and Kwilu), and by facility type (GRH and HC). Fisher’s exact test was used for bivariate comparisons of difference in the indicators between province and facility type. Missing data was ignored for the purposes of these simple, descriptive analyses. A majority of our expected cell counts were less than five; for continuity in the results, we used the more conservative estimate of Fisher’s exact test for the few relationships with expected cell counts greater than five. When cell count was greater than five, there was no change in significance between Pearson’s or Fisher’s exact test (data not shown). SAS Version 9.4 was used for all statistical analyses (SAS Institute Inc., Cary, NC).

## Results

### Facility condition

Among 72 health facilities, facility conditions were variable, as outlined in Table 1. Maternity wards (n=70, 97.2%) and delivery rooms (n=71, 98.6%) had the highest overall availability among the six rooms assessed per facility. This pattern was observed across both province and facility type. Among all facilities, 40.3% (n=29) had room for antenatal care, 46.5% (n=33) had room for labor, and 38.9% (n=28) had room for surgical intervention. Two-thirds of GRH facilities had room for labor (66.7%, n=8) and 42.4% (n=25) of HC facilities had room for labor. Room for surgical intervention was available in 55.6% (n=15) of facilities in Kwango, 28.9% (n=13) of facilities in Kwilu, 91.7% (n=11) of GRH facilities, and 28.3% (n=17) of HC facilities. There was a difference in the availability of rooms for surgical intervention by province (p<0.05) and facility type (p<0.001).

**Table 1.** Select indicators of facility conditions among 72 rural health facilities in the Democratic Republic of the Congo.

Indicators	All	Province		(P-value)	Facility Type		(P-value)
		Kwango (N=27)	Kwilu (N=45)		GRH ^1^ (N=12)	HC ^2^ (N=60)	
	N (%)	N (%)	N (%)		N (%)	N (%)	
**Infrastructure**							
Room for:							
Antenatal care	29 (40.3)	10 (37.0)	19 (42.2)	0.805	5 (58.3)	24 (40.0)	1.00
Consultation	37 (51.4)	16 (59.3)	21 (46.7)	0.338	5 (41.7)	32 (53.3)	0.537
Labor	33 (46.5)	9 (33.3)	24 (54.6)	0.093	8 (66.7)	25 (42.4)	0.203
Delivery	71 (98.6)	26 (96.3)	45 (100.0)	0.375	12 (100.0)	59 (98.3)	1.00
Surgical Intervention	28 (38.9)	15 (55.6)	13 (28.9)	0.045 *	11 (91.7)	17 (28.3)	<0.001 *
Maternity Wards	70 (97.2)	27 (100.0)	43 (95.6)	0.525	12 (100.0)	58 (96.7)	1.00
**Water and Electricity**							
Currently supplied with drinking water	18 (25.0)	4 (14.8)	14 (31.1)	0.164	5 (41.7)	13 (21.7)	0.160
Electricity available in the following rooms:							
Consultation	34 (49.3)	14 (56.0)	20 (45.5)	0.458	7 (58.3)	27 (47.4)	0.540
Labor	31 (49.2)	6 (31.6)	25 (56.8)	0.099	7 (63.6)	24 (46.2)	0.337
Delivery	48 (67.6)	17 (63.0)	31 (70.5)	0.604	10 (83.3)	38 (64.4)	0.314
Maternity	50 (69.4)	19 (70.4)	31 (68.9)	1.00	10 (83.3)	40 (66.7)	0.322
Operating	24 (37.5)	12 (54.6)	12 (28.6)	0.058	10 (83.3)	14 (26.9)	<0.001 *
**Equipment**							
Adult Weigh Scale	65 (90.3)	25 (92.6)	40 (88.9)	0.704	12 (100.0)	53 (83.3)	0.592
Delivery Table	65 (90.3)	25 (92.6)	40 (88.9)	0.704	12 (100.0)	53 (83.3)	0.592
Blood Pressure Device	58 (81.7)	22 (81.5)	36 (81.8)	1.00	11 (91.7)	47 (79.7)	0.444
Stethoscope	63 (97.5)	24 (88.9)	39 (86.7)	1.00	12 (100.0)	51 (85.0)	0.340
Fetoscope	70 (97.2)	26 (96.3)	44 (97.8)	1.00	12 (100.0)	58 (96.7)	1.00
Partogram	48 (66.7)	18 (66.7)	30 (66.7)	1.00	12 (100.0)	36 (60.0)	0.006 *
Forceps	9 (12.7)	6 (22.2)	3 (6.8)	0.075	6 (50.0)	3 (5.1)	<0.001 *
Vacuum Extractor	21 (29.2)	12 (44.4)	9 (20.0)	0.035 *	10 (83.3)	11 (18.3)	<0.001 *
Delivery Kit	58 (80.6)	23 (85.2)	35 (77.8)	0.547	11 (91.7)	47 (78.3)	0.439
Cesarean Section Kit	26 (36.1)	14 (51.9)	12 (26.7)	0.043 *	11 (91.7)	15 (25.0)	<0.001 *
Manual Intra-Uterine Suction Kit	8 (11.3)	3 (11.1)	5 (11.4)	1.00	7 (58.3)	1 (1.7)	<0.001 *
Newborn Suction Device	12 (16.7)	7 (25.9)	5 (11.1)	0.117	5 (41.7)	7 (11.7)	0.023 *
Manual Newborn Ventilation Kit	16 (22.5)	7 (25.9)	9 (20.4)	0.771	10 (83.3)	6 (10.2)	<0.001 *
Oxygen Bottle	5 (6.9)	3 (11.1)	2 (4.4)	0.357	2 (16.7)	3 (5.0)	0.191
Newborn Incubator	7 (9.7)	1 (3.7)	6 (13.3)	0.244	5 (41.7)	2 (3.3)	0.001 *
Kangaroo Mother Care Kit	8 (11.1)	8 (29.6)	0 (0.0)	<0.001 *	2 (16.7)	6 (10.0)	0.613
**Medicines and Supplies**							
Anti-convulsive (magnesium sulfate or diazepam)	54 (75.0)	23 (85.2)	31 (68.9)	0.164	9 (75.0)	45 (75.0)	1.00
Anti-hypertensive (hydralazine)	15 (20.8)	7 (25.9)	8 (17.8)	0.550	4 (33.3)	11 (18.3)	0.258
Iron-Folate	55 (76.4)	21 (77.8)	34 (75.6)	1.00	10 (83.3)	45 (75.0)	0.719
Ophthalmic antimicrobial (silver Nitrate 1%, tetracycline 1%)	46 (63.9)	21 (77.8)	25 (55.6)	0.077	9 (75.0)	37 (61.7)	0.517
Anesthetic (lidocaine or other)	45 (62.5)	20 (74.1)	25 (55.6)	0.138	9 (75.0)	36 (60.0)	0.515
Antibiotic (amoxicillin, Bactrim)	60 (83.3)	23 (85.2)	37 (82.2)	1.00	9 (75.0)	51 (85.0)	0.408
Oxytocic (Oxytocin)	62 (86.1)	24 (88.9)	38 (84.4)	0.733	10 (83.3)	52 (86.7)	0.669
Obstetrical gloves	58 (80.6)	21 (77.78)	37 (82.2)	0.761	10 (83.3)	48 (80.0)	1.00
Stitching materials	46 (63.9)	20 (74.1)	26 (57.8)	0.209	11 (91.7)	35 (58.3)	0.045 *
Antiseptic solution	57 (79.2)	23 (85.2)	34 (75.6)	0.384	10 (83.3)	47 (78.3)	1.00
Umbilical cord clamping materials	33 (45.8)	18 (66.7)	15 (33.3)	0.008 *	9 (75.0)	24 (40.0)	0.054

*p<0.05. ^1^GRH=Generalized reference hospital; ^2^HC=Health center

Facilities had low observed levels of drinking water supply overall, across province, and by facility type ( Table 1). There was a statistically significant difference in the availability of electricity to operating rooms by facility type (p<0.001). Electricity in operating rooms was available in 83.3% (n=10) of GRH facilities and 26.9% (n=14) of HC facilities.

Adult weigh scales, delivery tables, blood pressure devices, stethoscopes, fetoscopes, and delivery kits were available in frequencies greater than 75% both overall and across the two comparison groups ( Table 1). There was a statistically significant difference (p<0.05) between facility type in availability of partograms, forceps, vacuum extractors, cesarean section kits, manual intra-uterine suction kits, newborn suction devices, manual newborn ventilation kits, and newborn incubators ( Table 1). Partograms were available in 100% of GRH facilities (n=12) and 60% of HC facilities (n=36). Forceps were available in 50% (n=6) of GRH facilities and in 5.1% (n=3) of HC facilities. Vacuum extractors (83.3%, n=10), cesarean section kits (91.7%, n=11), and delivery kits (9.2%, n=11) were available in the highest frequency for GRH facilities. Kangaroo mother care kits were not available in any facilities in Kwilu. There was a significant difference (p<0.05) by province in the availability of vacuum extractors, cesarean section kits, and kangaroo mother care kits.

Umbilical-cord-clamping materials and anti-hypertensive medicines and supplies were available in the lowest frequencies both overall and across comparison groups. Availability of umbilical-cord-clamping materials was the only statistically significant difference by province (p<0.01); umbilical cord clamping materials were available in 33.3% (n=14) of facilities in Kwilu and in 40% of HC facilities (n=24). There was a statistically significant difference between stitching materials (p<0.05) by facility type. Stitching materials were available in 91.7% (n=11) of GRH facilities but in only 58.3% of HC facilities (n=35). Differences in umbilical cord clamping by facility type approached statistical significance (p=0.053).

### Obstetric and neonatal care practices

**Figure 2.**
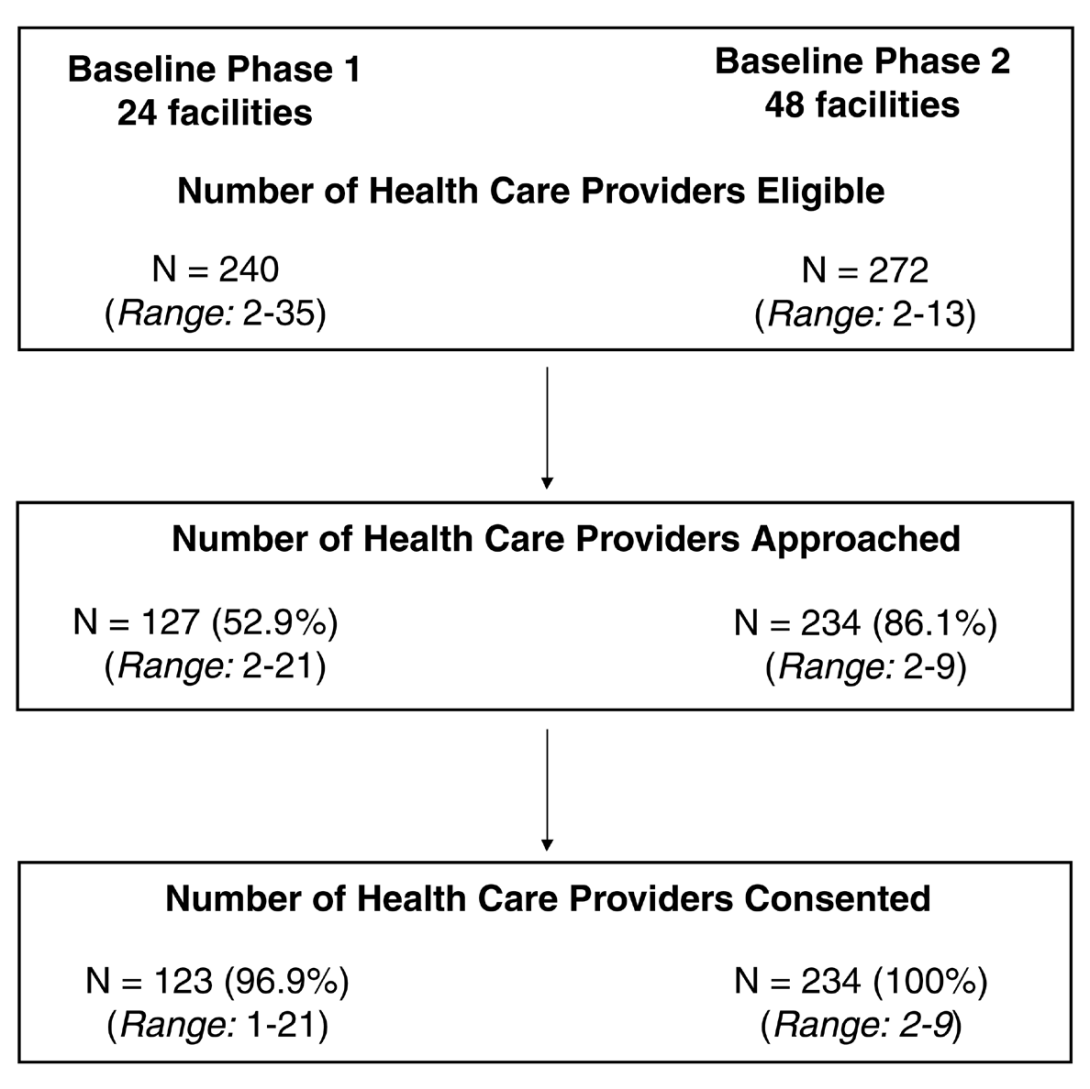
Recruitment of healthcare providers across baseline phase 1 and phase 2.

***Healthcare provider recruitment.*** Among the 24 facilities from the phase one survey, 240 health care providers were eligible, 127 (52.9%) were approached, and 123 (96.9%) consented to be observed for their routine MNH care practices ( Figure 2). Among the 48 facilities from the phase two survey, 272 health care providers were eligible, 234 (86.1%) were approached, and all health care providers approached consented to be observed (n=234, 100%). Common reasons that eligible participants were not approached were because they were sick or on leave from the facility at time of data collection (data not shown). We refer to these unique observations as provider-patient pairs.

***Antenatal care.*** An average of 10 provider-patient antenatal care pairs were observed per facility. A total of 740 observations of patient-provider antenatal care pairs were observed ( Table 2). A total of 269 (36.4%) provider-patient pairs were observed in Kwango, 471 (63.6%) in Kwilu, 127 (17.2%) in GRH facilities, and 613 (82.8%) in HC facilities. Blood pressure was measured in 94.9% (n=702) of all facilities and at a similar frequency across all comparison groups. Conversely, proteinuria was measured in 14.7% (n=109) of all observed provider-patient pairs. The highest frequency of screening for proteinuria occurred in GRH facilities (57.5%, n=73). There is a significant difference in HIV screening (p<0.0001), anemia screening (p<0.001), and blood pressure measurement (p<0.01) by province; one province was not consistently more likely to provide screening services than the other. There was a significant difference in all included screening measures and blood pressure measurement by facility type (all p<0.05, Table 2). Blood pressure measurement was 95.9% (n=114) in HC facilities and 89.8% (n=114) in GRH facilities; for all other screening tests, there were higher frequencies of observed provider-patient pair screenings in GRH facilities compared to HC facilities.

***Labor, delivery, and postnatal care.*** On average, two provider-patient pairs for labor, delivery, and postnatal care were observed per facility. A total of 136 labor, delivery, and postnatal care patient-provider pairs were observed ( Table 2). There were 54 (39.7%) facilities in Kwango, 82 (60.3%) in Kwilu, 23 (16.9%) in GRH facilities, and 113 (83.1%) in HC facilities. There was a statistically significant difference in documentation of stage of labor (p<0.001), women’s receipt of a uterotonic (p=0.005), and chlorohexidine applied to the umbilicus (p<0.05) by province. For all indicators, the frequency observed was higher in Kwango than Kwilu. There was a statistically significant difference in the use of a partogram to monitor labor by facility type (p<0.01). Progress of labor monitored by using a partogram and use of chlorohexidine on the umbilicus followed by dry cord care were observed in lower frequencies than other selected labor indicators. After delivery, mothers were provided with immediate postnatal care every 15 minutes in less than 50% of facilities both overall and across all groups; a range of 35.2% in Kwango to 46.3% in Kwilu.

**Table 2.** Select indicators of antenatal, labor, and postnatal care among 72 rural health facilities in the Democratic Republic of the Congo.

Indicators	All	Province		P-value	Facility Type		P-value
		Kwango	Kwilu		GRH ^1^	HC ^2^	
	N (%)	N (%)	N (%)		N (%)	N (%)	
**Antenatal Care**	**N=740 †**	**N=269 †**	**N=471 †**		**N=127 †**	**N=613 †**	
Anemia	468 (63.2)	114 (42.4)	354 (75.2)	<0.001 *	111 (87.4)	357 (58.2)	<0.001 *
Syphilis	117 (15.8)	43 (6.0)	74 (15.7)	0.917	59 (46.5)	58 (9.5)	<0.001 *
HIV	453 (61.2)	234 (67.0)	219 (46.5)	<0.001 *	117 (92.1)	336 (54.8)	<0.001 *
Proteinuria	109 (14.7)	37 (13.8)	72 (15.3)	0.592	73 (57.5)	35 (5.9)	<0.001 *
Blood Pressure measured	702 (94.9)	247 (91.8)	455 (96.6)	0.009 *	114 (89.8)	588 (95.9)	0.008 *
**Labor and Delivery**	**N=136 †**	**N=54 †**	**N=82 †**		**N=23 †**	**N=113 †**	
Stage of labor is documented	106 (77.9)	51 (94.4)	55 (67.1)	<0.001 *	21 (91.3)	85 (75.2)	0.105
Progress of Labor monitored with partogram	52 (38.2)	19 (35.2)	33 (40.2)	0.592	16 (69.6)	36 (31.9)	0.002 *
Woman receives uterotonic immediately after birth	113 (83.1)	51 (94.4)	62 (75.6)	0.005 *	21 (91.3)	92 (81.4)	0.364
Immediate Newborn Care:							
Newborn is dried immediately and thoroughly	135 (99.3)	54 (100.0)	81 (98.8)	1.00	23 (100.0)	112 (99.1)	1.00
Spontaneous breathing assessed at birth	134 (98.5)	54 (100.0)	80 (94.6)	0.518	23 (100.0)	111 (98.2)	1.00
Cord is clamped and cut within 1-3 minutes	128 (94.1)	53 (98.2)	75 (91.5)	0.145	23 (100.0)	105 (92.9)	0.351
7.1% Chlorohexidine is applied to the umbilicus followed by dry cord care	24 (17.7)	15 (27.8)	9 (11.0)	0.020 *	7 (30.4)	17 (15.0)	0.128
Baby has skin to skin contact with mother for at least 30 minutes	99 (72.3)	41 (75.9)	58 (70.7)	0.559	16 (69.6)	83 (73.5)	0.798
**Postnatal Care**							
Immediate postnatal care is provided to mothers every 15 minutes during the first two hours	57 (41.9)	19 (35.2)	38 (46.3)	0.218	10 (43.5)	47 (41.6)	1.00

*p <.05; ^1^GRH=Generalized Reference Hospital, ^2^HC=Health center †Number of observed provider-patient pairs

### Emergency obstetric and newborn care (EmONC)

**Figure 3.**
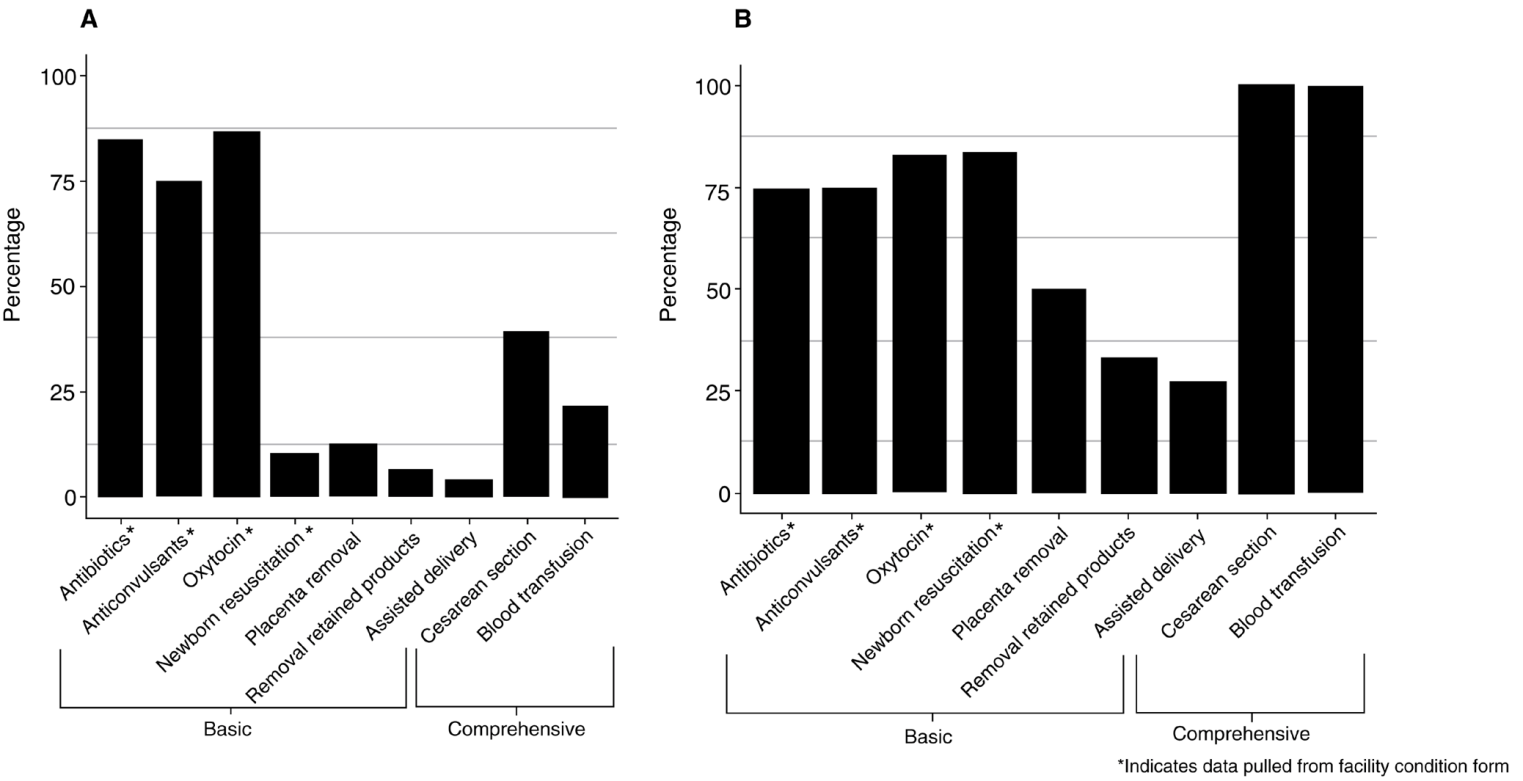
7–9 EmONC signal functions for HC ( **A**) and GRH ( **B**) facilities.

Antibiotics, anticonvulsants, and oxytocin were available in more than 68.9% of facilities both overall and across province or facility type ( Table 3, Figure 3). Availability of newborn resuscitation and whether placenta removal, removal of retained products, assisted delivery, cesarean sections, and blood transfusions were performed in the past 3 months in facility are shown as percentages among health facilities where information was available. Both Kwilu and HC facilities were missing one facility for manual newborn resuscitation kits and cesarean sections. These were two, different facilities but were both from the same health zone. Information was missing for all groups on manual removal of the placenta, removal of retained placenta products, assisted deliveries, and blood transfusions. When information was available, GRH facilities had the highest frequency of availability of newborn ventilation kits (83.3%, n=10), cesarean sections (100%, n=12), and blood transfusions (100%, n=8). Manual removal of placenta took place in 82.5% (n=33) of all facilities and 50% (n=3) of GRH facilities.

**Table 3.** Emergency obstetric and neonatal care (EmONC) signal functions among 72 rural health facilities in the Democratic Republic of the Congo.

Signal Functions	All	Province		Facility Type	
		Kwango (N=27)	Kwilu (N=45)	GRH ^1^ (N=12)	HC ^2^ (N=60)
	N (%)	N (%)	N (%)	N (%)	N (%)
Antibiotics available					
Yes	60 (83.3)	23 (85.2)	37 (82.2)	9 (75.0)	51 (85.0)
No	12 (16.7)	4 (14.8)	8 (17.8)	3 (25.0)	9 (15.0)
Anticonvulsants available					
Yes	54 (75.0)	23 (85.2)	31 (68.9)	9 (75.0)	45 (75.0)
No	18 (25.0)	4 (14.8)	14 (31.1)	3 (25.0)	15 (25.0)
Oxytocin Available					
Yes	62 (86.1)	24 (88.9)	38 (84.4)	10 (83.3)	52 (86.7)
No	10 (13.9)	3 (11.1)	7 (15.6)	2 (16.7)	8 (13.3)
Manual Newborn Resuscitation Kit					
Yes	16 (22.2)	7 (25.9)	9 (20.45)	10 (83.3)	6 (10.2)
No	55 (76.4)	20 (74.1)	35 (79.5)	2 (16.7)	53(89.8)
Missing	1	0	1	0	1
Manual Removal of Placenta					
Yes	33 (82.5)	2 (33.3)	5 (15.2)	3 (50.0)	4 (12.1)
No	7 (17.5)	4 (66.7)	28 (84.8)	3 (50.0)	29 (87.9)
Missing	32	21	12	6	27
Removal of retained placenta products					
Yes	4 (10.8)	2 (33.3)	5 (15.2)	2 (33.3)	2 (6.5)
No	33 (89.2)	4 (66.7)	28 (84.8)	4 (66.7)	29 (93.5)
Missing	35	21	12	6	29
Assisted Delivery					
Yes	5 (8.3)	3 (15.8)	2 (6.5)	3 (27.3)	2 (4.8)
No	55 (91.7)	16 (84.2)	29 (93.5)	8 (72.7)	47 (95.9)
Missing	12	8	14	1	11
Cesarean section					
Yes	35 (49.3)	12 (44.4)	20 (45.5)	12 (100.0)	23 (39.0)
No	36 (50.7)	15 (55.6)	24 (54.5)	0 (0.0)	36 (61.0)
Missing	1	0	1	0	1
Woman received blood transfusion					
Yes	16 (35.6)	6 (60.0)	10 (28.6)	8 (100.0)	8 (21.6)
No	29 (64.4)	4 (40.0)	25 (71.4)	0 (0.0)	29 (78.4)
Missing	27	17	10	4	23

^1^GRH=Generalized reference hospital; ^2^HC=Health center.

**Figure 4.**
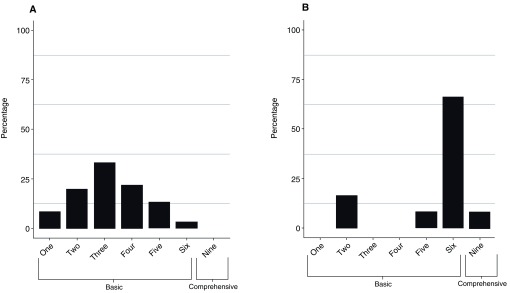
Sum of EmONC signal functions for HC ( **A**) and GRH ( **B**) facilities.

When the EmONC signal functions were summed, only one GRH facility was able to provide all nine CEmONC signal functions ( Figure 4). Two-thirds (66.7%, n=8) of GRH facilities provide six signal functions. Among the facilities that provide six signal functions, all provide antibiotics, anticonvulsants, oxytocin, and cesarean sections (see Supplemental Table 6 for additional patterns of availability). Among HC facilities, 74.7% were able to provide two to four signal functions. The most common signal functions that were not provided by the HC facilities were placenta removal, removal of retained products, cesarean sections, or blood transfusions. In these cases, signal functions were observed in low frequency and had high values of missing data ( Table 3). For example, placenta removal was not observed in 29 (93.5%) of HC facilities where information was available and information on placenta removal was missing for 29 of a total 60 HC facilities.

## Discussion

The findings from the present study highlight some key profiles of the current facility conditions, routine obstetric and neonatal care practices, as well as availability and/or use of emergency obstetric and neonatal care (EmONC) among a sample of 72 rural health facilities in the Kwango and Kwilu provinces, DRC. EmONC indicators were developed to identify key factors that can help to reduce maternal and perinatal mortality. Previous studies have highlighted the impact of EmONC to reduce maternal and perinatal deaths in high risk pregnancies ^12, 13^. In general, the results of our baseline analysis show that facility conditions are poor and lack sufficient medicines and supplies. Few facilities are supplied with drinking water and the availability of electricity is variable. Despite these poor facility conditions, we observed that GRH and HC facilities in rural DRC have higher than expected proportions of the key 7–9 EmONC signal functions. While only one facility is providing the full capacity of EmONC care according to what is expected—one GRH facility is providing all nine EmONC signal functions—most GRH facilities are providing six EmONC signal functions. There is more of a range in the EmONC functions provided by HC facilities, with most providing between and four signal functions out of an expected seven. Antibiotics, anticonvulsants, and oxytocin are available in high frequencies. Frequency of newborn resuscitation, placenta removal, removal of retained products, and assisted delivery were much lower. However, this may be attributed to both the large number of missing data during the data collection phase as well as the missing supplies need to perform the functions for placenta removal and removal of retained products. A majority of the missing data in the three-month delivery record for these two variables is when there are no supplies to perform the procedure (e.g., out of 33 missing observations for removal of the placenta, 28 were from a not available manual intrauterine suction kit). These results are consistent with the other reports indicating great variability in availability of EmONC functions across health facilities in Lumbashi, a southern province in the DRC, as well as other African countries ^14– 16^. In Lumbashi, EmONC availability fell short with one facility providing comprehensive care, all facilities providing at least one out of nine functions, and assisted vaginal delivery was the least performed function overall ^15^.

The current MNH providers’ skills, knowledge and attitude were evaluated by observing their routine practices during antenatal, labor, delivery, and postnatal care ( Table 2). There is variability in the spread of the antenatal care indicators. For example, the distribution of blood pressure measures is positively skewed towards being observed in all antenatal care observations while HIV and anemia screening have bimodal distributions, with screening for HIV and anemia being largely never or always observed (data not shown). Therefore, the bivariate comparison results should be interpreted with some caution, considering the large degree of variability across facilities for antenatal care screening. Additionally, we found differences in screening rates both by province and facility type, with higher observed screening practices in the GRH compared to the HC facilities and no distinct pattern to screening by province. Our study design does not allow us to know which antenatal care visit we are observing (e.g., the first, the second, or the third). Given that HIV and syphilis testing are not expected to be performed at each antenatal care visit, the expected frequency is not 100%. However, we would expect that the frequency of HIV and syphilis screenings would be similar if both screening practices were integrated into health care practices. Our results show that there are differences in the frequency of syphilis and HIV screening, which suggests that these two screenings are not integrated into the health care system.

Basic components of labor and delivery care were observed in high frequencies (newborn dried immediately and thoroughly, spontaneous breathing assessed at birth, and cord clamped and cut within one to three minutes). However, components of labor and delivery that may require more knowledge were observed in lower frequencies. For example, the use of a partogram to assess the stage of labor was below 40.2% in all groups, except for in GRH facilities, where 69.2% of observed provider-patient pairs used a partogram to monitor labor and delivery. The infrastructure data shows that partograms were available in two-thirds of all facilities. This suggests that while partograms were available, they are not being used. Further, it was noted qualitatively by data collectors that partograms were not filled correctly or that providers did not use partograms correctly (data not shown). Similarly, the application of chlorohexidine to the umbilicus and dry cord care was observed in a low proportion of provider-patient pairs despite antiseptics being available in 79.2% of all facilities. Additionally, secondary care, such as the baby having skin-to-skin contact with the mother or frequency of postnatal care provided to the mother, have room for improvement. Overall, these findings provide the baseline information on MNH providers’ skills, knowledge, and attitude to evidence-based antenatal, labor and delivery, postnatal care, and other supportive care. The observed inconsistent and insufficient provisions of antenatal, labor, delivery, and postnatal care across province and health facilities clearly point out the needs for designing, developing, and implementing a clinical mentoring program aimed to improve quality of MNH care in the health facilities.

There were fewer differences in terms of facility conditions, obstetric and neonatal care practices, and availability and/or use of emergency of obstetric and neonatal care (EmONC) between Kwango and Kwilu provinces. As expected, we did find that GRHs, overall, had better facility conditions, obstetric and neonatal care, and availability of EmONC than HC facilities. Forceps and vacuum extractors were available in low frequencies overall, but the lowest frequency was observed in HC facilities. This may directly contribute to the lower rates of assisted deliveries in the past three months among HC facilities. If neither of the tools were available to assist with deliveries, then assisted deliveries were likely not performed. Comprehensive care is theoretically only available in GRH facilities. Cesarean sections took place in all the GRH facilities. Interestingly, cesarean sections were recorded in 39.0% (n=23) of HC facilities. There was more missing information on women’s receipt of blood transfusions. Among facilities where information was available, all GRH facilities performed blood transfusions (n=8) in the last three months. However, blood transfusions were noted in less than a quarter of HC facilities (21.6%, n=8). While we do not have indicators of quality of care, this data points to the fact that health care providers are attempting to provide a range of comprehensive care to patients.

The high frequency of cesarean sections in HC facilities is also interesting, considering that there is a difference in the availability of stitching materials, cesarean section kits, room for surgical intervention, and electricity in operating rooms by facility type. HC facilities have a lower frequency of both materials than GRH facilities. GRH facilities are structured to have doctors on staff to perform cesarean sections and blood transfusions. Our observations confirmed every GRH facility had a medical doctor to perform cesarean sections; a medical doctor was observed in 18 HC facilities. Among these 18 HC with a medical doctor, 13 HC facilities are providing cesarean sections, leaving ten facilities providing cesarean sections without a medical doctor on staff (data not shown). It may be that other staff (e.g., midwives or A1–A3 nurses) at the HC facilities are performing cesarean sections. Additional data is needed to better understand this relationship and its impact on maternal and neonatal mortality.

This study has provided a wealth of information on the current facility conditions and baseline data on MNH providers’ routine care practices in a rural area of the DRC. Further, the data was collected using the ODK system, which allowed for quick and accurate transfer of data from the rural facilities in the DRC to the Tulane Study Coordinating Center in New Orleans, USA. As such, we were able to avoid issues transferring paper data copies between multiple coordinators, issues of deciphering hand-written notes, or manual data entry errors. We provided data collectors with motorbikes, so they were able to move with more ease between health facilities independent of road conditions, which often worsened during the rainy season. We also used an extensive quality control system with people working at multiple points in the pipeline both in the DRC and in New Orleans, USA. Quality control was ensured throughout the baseline data collection process through a series of regular meetings; in addition to the regular skype meetings, the Kinshasa team met regularly with the DRC Ministry of Health and UNICEF members. This allowed for issues to be addressed as they arose, which enhanced our ability to collect data quickly and accurately.

Our study has several limitations. First, data collectors only observed health care providers’ routine MNH practices. While we have measures of availability and performance of evidence-based MNH care or interventions to patients based on EMEN criteria, we did not measure whether the health care providers performed the care correctly or sufficiently. For example, our data collectors observed whether a partogram was available and if it was used to monitor the progress of labor at the facility level but did not quantitatively assess if the partogram was filled correctly. While some qualitative observations were noted by data collectors regarding appropriate use, this was not done systematically. Additionally, our data collectors may not have had the expertise or capability to judge whether a partogram was filled appropriately. A second consequence of our observation approach is that our measures of care may be overestimations. Healthcare providers may be practicing a higher frequency or quality of care while being observed by the data collectors than what is provided normally. Second, information for the EmONC signal functions was pulled from two sources: the availability of medicines and supplies and review of the past 3-month delivery record. It was qualitatively noted by some data collectors that the three-month delivery record was not maintained adequately, which could have resulted in missing information or in underreporting of certain outcomes. Further, availability of medicine or tools used to implement emergency obstetric care does not mean that it was used nor if it was used appropriately ^11^. These issues may contribute to the lower frequencies of newborn resuscitation kits, placenta removal, removal of retained products, and assisted deliveries. Third, there were large amounts of missing data in our results. Due to the cross-sectional and observational nature of our data collection process, we chose to ignore missing data in our bivariate comparisons. Due to the large amount of missing data, this may result in a type 2 error, causing us to not observe differences when they actually occur. Fourth, our metric for this paper of availability of medicine or equipment doesn’t differentiate whether those medicines or drug are valid or expired and whether the equipment is functional or not (raw data shown in Supplementary Table 3). Finally, the facilities included in this report were not randomly selected but were selected based on administrative criteria for facilitating the implementation of clinical mentoring program. The data presented in this report thus may not represent all facilities in the Kwango and Kwilu provinces. However, we do have data from the 48 facilities removed after phase one of the survey. Future research may explore the characteristics of all facilities where baseline data was collected for a more robust picture of rural health facilities in the DRC. To expand on these results, future research should explore the quality of EmONC function beyond availability and functionality; this may completed through a systematic qualitative assessment which has been done in previously in Lumbashi ^15^. Future research may also explore the capacity of HC facilities in providing comprehensive care (e.g., distance from GRH facilities or staffing).

## Conclusion

Despite poor facility conditions and lack of supplies, GRH and HC facilities were able to provide essential maternal and neonatal care in the rural provinces of the Democratic Republic of the Congo. The observed inconsistent and insufficient provisions of antenatal, labor, delivery, and postnatal care across province and health facilities clearly point out the needs for developing and implementing a clinical mentoring program aimed to improve quality of MNH care in the health facilities. The findings from this baseline survey can be used to guide the provision of essential needs (e.g., equipment, medicine, structure) to the health facilities for better delivery of maternal and neonatal care.

## Data availability

### Underlying data

Source data examined in this study, available in xlsx, are available on OSF. DOI: http://doi.org/10.17605/OSF.IO/S5QJ7 ^17^.

### Extended data

Supplemental tables associated with this study are available on OSF. DOI: http://doi.org/10.17605/OSF.IO/S5QJ7 ^17^.

**Supplemental Table 1. Baseline facility condition assessment form.**

**Supplemental Table 2. Baseline questionnaire for antenatal, labor, delivery and postnatal care.**

**Supplemental Table 3. Three-month delivery record review and extraction form.**

**Supplemental Table 4. Availability and functionality of equipment, medicines, and supplies.**

**Supplemental Table 5. Data sources for EmONC signal functions.**

**Supplemental Table 6. Sum of EmONC signal functions by facility type with availability of key functions qualitatively identified.**

Data available under the terms of the Creative Commons Zero "No rights reserved" data waiver (CCO 1.0 Public domain dedication).
