# Supplementary material for: Facility conditions, obstetric and neonatal care practices, and availability of emergency obstetric and neonatal care in 72 rural health facilities in the Democratic Republic of the Congo: A cross-sectional study
Source: Gates Open Res. 2019 Jul 23;3:13. Originally published 2019 Feb 5. [Version 2] doi: 10.12688/gatesopenres.12905.2 (PMC6676177; doi:10.12688/gatesopenres.12905.2)
Supplement: Peer review history

# Peer review: Reviewer response for version 2

**DOI:** 10.21956/gatesopenres.14171.r27569

**Published:** 2019-07-31

**Authors:** Wei-Hong Zhang

**Affiliations:** 1 Department of Public Health and Primary Care, International Centre for Reproductive Health (ICRH), Ghent University, Ghent, Belgium

I am satisfied with responses from authors, point by point in a clear way, and amended correctly in the new version.

 I have no future comment and agree for accepting indexing.

I confirm that I have read this submission and believe that I have an appropriate level of expertise to confirm that it is of an acceptable scientific standard.

# Peer review: Reviewer response for version 1

**DOI:** 10.21956/gatesopenres.14001.r27082

**Published:** 2019-07-08

**Authors:** Wei-Hong Zhang

**Affiliations:** 1 Department of Public Health and Primary Care, International Centre for Reproductive Health (ICRH), Ghent University, Ghent, Belgium

General comment:

- This paper presents well and provided a wealth of information on the current facility conditions and baseline data on MNH providers’ routine care practices in a rural area of the DRC. Objectives, methodology and the results are all aligned well.

In the Methods section: 

1. The presentation about the facilities included in the survey in two phases were not clear (figure 1), more particularly, about the 48 facilities which did not meet the criteria; more info about those 48 would be useful. In addition, how many eligible facilities? Among them, what number of facilities, if there were any, refused to participate? What were the methods used for motiving their participation if there were any?
2. Why, after excluding those 48 facilities, were there still 48 facilities included in the survey? (72-48 = 24 facilities) (figure 1). 

In the Results section:

1. Table 1: Comparison about the indicators of facility conditions. What about the socioeconomic conditions among those compared facilities? Were they similar or different? What was the size of the population?
2. There were no comparisons on the facility conditions between the pre-defined intervention group (48) and control group (24), why? Were the facility conditions comparable between two groups?
3. Figure 2: The size of the health providers varied largely among the facilities, this should be taken into account in the analysis as confounding factors.
4. Table 3: For the variable of Assisted delivery, comparison between HC, the total does not equal 100%.

In the Discussion:

- No comparison between the results of this study with others. More literature review would be useful?

I confirm that I have read this submission and believe that I have an appropriate level of expertise to confirm that it is of an acceptable scientific standard, however I have significant reservations, as outlined above.

# Response

**Published:** 2019-07-16

**Authors:** Xu Xiong

**Affiliations:** Tulane University School of Public Health and Trop, USA

*The presentation about the facilities included in the survey in two phases were not clear (figure 1), more particularly, about the 48 facilities which did not meet the criteria; more info about those 48 would be useful. In addition, how many eligible facilities? Among them, what number of facilities, if there were any, refused to participate? What were the methods used for motiving their participation if there were any.*

- More details about facility eligibility and inclusion of the 72 facilities was added to the updated version.
- The two phases have been clarified in the text with the following:
  - “For the present analysis of the baseline survey data, the final 72 facilities were a combination of two phases of baseline data collection. The first phase of data collection was conducted June-September 2017 and the second phase of data collection was conducted from December 2017-May 2018. Forty-eight facilities were removed by UNICEF and the DRC Ministry of Health after baseline Phase 1 because they did not meet the first administrative criteria, of more than 200 annual deliveries. Baseline Phase 2 was conducted to replace these 48 facilities with 48 new facilities to have a total of 72 facilities that are included for the ongoing clinical mentoring project (Figure 1).” 
- The facilities included for the baseline surveys were selected by the DRC Ministry of Health to facilitate the implementation of the ongoing clinical mentorship program, which is considered as a national MCH program in the DRC. To our knowledge, no facility refused to participate. The final 72 participating facilities receive some essential equipment upgrades and consumables through the UNICEF and World Bank support schemes.

*Why, after excluding those 48 facilities, were there still 48 facilities included in the survey? (72-48 = 24 facilities) (figure 1). *

- This has been updated in the text (see our response to above question for the reason excluding the 48 facilities after baseline Phase 1) and in the Figure 1 to clarify confusion about Baseline Phases 1 and 2.

*Table 1: Comparison about the indicators of facility conditions. What about the socioeconomic conditions among those compared facilities? Were they similar or different? What was the size of the population?*

- We collected the indicators to assess the facility conditions (e.g., availability of rooms, equipment, medicines/drugs, and supplies) in each health facility. These indicators may be the proxies for “socioeconomic conditions” among the facilities. We did not collect data on socioeconomic status among health providers and patients in each of the facilities. We do not have the information on the size of the populations served by the facilities.

*There were no comparisons on the facility conditions between the pre-defined intervention group (48) and control group (24), why? Were the facility conditions comparable between two groups?*

- The main purpose of this paper is to have baseline information on the facility condition of the 72 facilities, including the 48 intervention and 24 controls. At this stage of the study, there is no intention to compare these two groups. After completing the ongoing clinical mentorship (intervention) program, an endline survey will be conducted on the same 72 facilities. We will then perform the comparisons between 48 intervention facilities and 24 control groups to assess the effectiveness of the clinical mentorship program. The difference in the facility conditions between these two groups will be compared and be considered as a potential confounder to adjust.  

*Figure 2: The size of the health providers varied largely among the facilities, this should be taken into account in the analysis as confounding factors.*

- This point is very well taken, we will take this into account when doing a multivariate analysis in the final paper comparing the effectiveness of the study overall.

*Table 3: For the variable of Assisted delivery, comparison between HC, the total does not equal 100%.*

- This has been corrected in Table 3.

*No comparison between the results of this study with others. More literature review would be useful?*

- The results presented in this paper mainly reflect the baseline information, including variables for measuring healthcare providers’ skills, knowledge and attitudes during their routine MNH care, as well as on facility conditions. These variables will be used to assess the effectiveness of the ongoing clinical mentorship project being implemented in the DRC. Few previous studies reported this information. We added the following statements comparing availability of EmONC functions from our study with others:
  - “These (our) results are consistent with the other reports indicating great variability in availability of EmONC functions across health facilities in Lumbashi, a southern province in the DRC, as well as other African countries ^14-16^. In Lumbashi, EmONC availability fell short with one facility providing comprehensive care, all facilities providing at least one out of nine functions, and assisted vaginal delivery was the least performed function overall ^15^.” 

# Peer review: Reviewer response for version 1

**DOI:** 10.21956/gatesopenres.14001.r27148

**Published:** 2019-05-08

**Authors:** Linnea Zimmerman

**Affiliations:** 1 Johns Hopkins Bloomberg School of Public Health, Baltimore, MD, USA

This paper reviews baseline data from an ongoing program in two provinces in DRC. The objective of this paper is to describe the current facility conditions, obstetric and neonatal care practices (skills, knowledge, and attitudes), and availability and use of emergency obstetric and neonatal care (EmONC) among a sample of 72 rural health facilities in the Kwango and Kwilu provinces, DRC. Overall, the authors do a good job synthesizing a large amount of data. Some additional clarification is needed in the methods section and a few minor language changes would improve clarity. Overall, the paper is well-written and with mostly minor revisions, is worthy of indexing. 

**Comments:**

- What is the difference between Baseline Phase 1 and Baseline Phase 2? 1-2 sentences explaining that difference would be useful. Explaining this difference and how it affected the sample would provide clarification that would likely answer some of the questions below.
- The selection diagram was not very clear to me initially. I did not understand how the removal of 48 facilities from 72 resulted in 48 facilities. Later, it is more clear that the 24 and 48 were combined for 72 facilities. Addressing the comment above and adding slightly more detail in the written description would be useful.
- Were the data collectors trained health professionals? Observation generally requires more skills than typical face to face interviews. More detail to clarify if these are health professionals with a working knowledge of delivery care procedures would be useful, especially as it relates to the mention in the limitations section that they may not have had the expertise to judge whether procedures were correctly followed.
- Page 4 – item 2: Why were no postnatal care visits observed? As the checklists specifically mention postnatal care, why was baseline data assessing postnatal care not gathered? Is postnatal care not a part of the intervention?
- The statement that all eligible providers were approached for the survey is at odds with Figure 2, where 52.9 and 86.1 percent of providers were approached. Please explain why not all eligible providers were approached in the Data Collection section.
- Page 5 – Analysis: Specify how you treat missing data, given the large percentage of missing data for some indicators. 
- Limitations – there is a significant amount of missing data for several indicators. Note this in the limitations and what this missing data might mean for your results and interpretation.

 

**Minor:**

- Change “neonatal mortality rates” to rate on page 3, paragraph 2.
- What does “also” in the sentence “The DRC boasts one of the highest rates of institutional deliveries in sub-Saharan Africa (80%), with eight out of every ten births also assisted by a skilled provider.” refer to? Unclear what it is adding onto.
- Suggest to rephrase the second part of the sentence in “There is a significant difference in HIV screening (p<0.0001), anemia screening (p<0.001), and blood pressure measurement (p<0.01) by province; there is no consistency in observed provider-patient pair screenings by province” to clarify that you mean that one region was not consistently more likely to provide services than the other. 
- “Both Kwilu and HC facilities were missing one facility for manual newborn resuscitation kits and cesarean sections.” Is this the same facility? If the same, suggest rephrasing to say “one Kwilu HC facility was missing … ”
- “However, this may be attributed to the large number of missing data for placenta removal and removal of retained products as well as the missing supplies needed to perform each of these functions.” Are the data for the supplies missing from the dataset or not available at the facility? May be useful to refer to supplies as not available if the latter.

I confirm that I have read this submission and believe that I have an appropriate level of expertise to confirm that it is of an acceptable scientific standard.

# Response

**Published:** 2019-07-16

**Authors:** Xu Xiong

**Affiliations:** Tulane University School of Public Health and Trop, USA

*What is the difference between Baseline Phase 1 and Baseline Phase 2? 1-2 sentences explaining that difference would be useful. Explaining this difference and how it affected the sample would provide clarification that would likely answer some of the questions below.*

 

- The text has been updated to provide clarity regarding the reason behind the two phases of baseline data collection. The following has been added to the text:
  - “For the present analysis of the baseline survey data, the final 72 facilities were a combination of two phases of baseline data collection. The first phase of data collection was conducted June-September 2017 and the second phase of data collection was conducted from December 2017-May 2018. Forty-eight facilities were removed by UNICEF and the DRC Ministry of Health after baseline Phase 1 because they did not meet the first administrative criteria of more than 200 annual deliveries. Baseline Phase 2 was conducted to replace these 48 facilities with 48 new facilities to have a total of 72 facilities that are included for the ongoing clinical mentoring project (Figure 1).”

*The selection diagram was not very clear to me initially. I did not understand how the removal of 48 facilities from 72 resulted in 48 facilities. Later, it is more clear that the 24 and 48 were combined for 72 facilities. Addressing the comment above and adding slightly more detail in the written description would be useful.*

- The headers for each section of the diagram have been updated for clarity. In addition, the text has been updated to further help clarify the two baseline phases.

*Were the data collectors trained health professionals? Observation generally requires more skills than typical face to face interviews. More detail to clarify if these are health professionals with a working knowledge of delivery care procedures would be useful, especially as it relates to the mention in the limitations section that they may not have had the expertise to judge whether procedures were correctly followed.*

- This criticism is well taken and the text has been updated to reflect the qualifications of data collectors. This update can be found in the first line of the first paragraph under Methods—Data collection.

*Page 4 – item 2: Why were no postnatal care visits observed? As the checklists specifically mention postnatal care, why was baseline data assessing postnatal care not gathered? Is postnatal care not a part of the intervention?*

- Postnatal care visits were observed - an expected 2 for each facility. The raw data is available in the data repository. The data presented in this report is selected “key variables” that are presented. Table 2 outlines 5 measures for antenatal care, 9 for labor and delivery, and one for postnatal care.

*The statement that all eligible providers were approached for the survey is at odds with Figure 2, where 52.9 and 86.1 percent of providers were approached. Please explain why not all eligible providers were approached in the Data Collection section.*

- This confusion is noted and has been corrected by two changes:
  - One line in data collection clarifying that all health care providers who perform maternal and neonatal care are eligible, but only those who were present at time of data collection were approached.
  -  A line was added to the results to explain the discrepancy noted qualitatively by data collectors.

*Page 5 – Analysis: Specify how you treat missing data, given the large percentage of missing data for some indicators.*

- This is a point well taken and has been added to the text in the last line of the Statistical Analysis: Analysis section.

*Limitations – there is a significant amount of missing data for several indicators. Note this in the limitations and what this missing data might mean for your results and interpretation.*

- This is a point well taken and has been added to the text as a third limitation.

*Change “neonatal mortality rates” to rate on page 3, paragraph 2.*

- This was changed in the text.

*What does “also” in the sentence “The DRC boasts one of the highest rates of institutional deliveries in sub-Saharan Africa (80%), with eight out of every ten births also assisted by a skilled provider.” refer to? Unclear what it is adding onto.*

- This was changed to reflect that it means that both 80% of deliveries occur in institutions and 8/10 are assisted by a skilled birth attendant. The following was added to the text:
  - “The Democratic Republic of the Congo (DRC) boasts one of the highest rates of institutional deliveries in sub-Saharan Africa (80%). Additionally, eight out of every ten births is assisted by a skilled provider [15]. However, the maternal mortality ratio and neonatal mortality in the DRC are still among the highest in the world (846 maternal deaths/100,000 live births and 28 newborn deaths/1,000 live births) [2, 15], contributing to 4% of global newborn deaths [15, 16]. Taken together, this suggests that while the availability of healthcare workers is high, there may be issues with the quality of care provided. Therefore, there is a need for continued education for health care providers.”

*Suggest to rephrase the second part of the sentence in “There is a significant difference in HIV screening (p<0.0001), anemia screening (p<0.001), and blood pressure measurement (p<0.01) by province; there is no consistency in observed provider-patient pair screenings by province” to clarify that you mean that one region was not consistently more likely to provide services than the other. *

- This has been changed in the text.

*“Both Kwilu and HC facilities were missing one facility for manual newborn resuscitation kits and cesarean sections.” Is this the same facility? If the same, suggest rephrasing to say “one Kwilu HC facility was missing … *

- These are different facilities and we acknowledge the confusion of this phrasing. We have changed this line in the text.

*“However, this may be attributed to the large number of missing data for placenta removal and removal of retained products as well as the missing supplies needed to perform each of these functions.” Are the data for the supplies missing from the dataset or not available at the facility? May be useful to refer to supplies as not available if the latter.*

- This criticism is well taken and has been clarified in the text. There is missing in both, but largely in supplies. An example has been provided for clarity as well.
